# Supplementary material for: Economic freedom index effects on inbound tourism in European countries: A spatial analysis
Source: PLoS One. 2025 Apr 1;20(4):e0316133. doi: 10.1371/journal.pone.0316133 (PMC11960978; doi:10.1371/journal.pone.0316133)
Supplement: Attachment — Submitted filename: PONE-D-23-19081 with comments.pdf [file pone.0316133.s002.pdf]

PLOS ONE

Economic Freedom Index Effects on Tourist’s Arrivals in European Countries: A Spatial Panel Analysis

--Manuscript Draft--

Manuscript Number: PONE-D-23-19081

Article Type: Research Article

Full Title: Economic Freedom Index Effects on Tourist’s Arrivals i[illegible]uropean Countries: A Spatial Panel Analysis

Short Title: Economic Freedom Index Effects on Tourist’s Arrivals

Corresponding Author: Sakar Hasan Hasan Hamza, Phd student
Beijing Institute of Technology
Bei Jing Shi, CHINA

Keywords: Spatial panel analysis; Economic freedom; European countries; Tourism

Abstract: This paper assesses the impact of economic freedom index components on tourist arrivals in 41 European countries, the world's leading tourism destinations, between 2005 and 2018. We are the first to use a spatial panel data method and consider spatial spillover to investigate the mentioned relationship. We find an increase in neighboring countries' GDP per capita results in higher domestic tourist arrivals[illegible] Additionally, results show an increase in neighboring countries' prices increases domestic tourists’ arrivals. More importantly, the economic freedom index has a significant effect on tourism arrivals in Europe. While some components of economic freedom have marginal effects, the coefficients for the Trade Freedom Index, Investment Freedom Index, Property Rights Index, and Government Integrity Index are significant and positive. Our findings have meaningful implications for policymakers interested in sustainable tourism in European countries.

Order of Authors: Sakar Hasan Hasan Hamza, Phd student

Additional Information:

Question	Response
**Financial Disclosure**<br><br>Enter a financial disclosure statement that describes the sources of funding for the work included in this submission. Review the submission guidelines for detailed requirements. View published research articles from *PLOS ONE* for specific examples.<br><br>This statement is required for submission and **will appear in the published article** if the submission is accepted. Please make sure it is accurate.	The author(s) received no specific funding for this work.

**Unfunded studies**<br>Enter: *The author(s) received no specific funding for this work.*<br><br>**Funded studies**<br>Enter a statement with the following details:<br>• Initials of the authors who received each award<br>• Grant numbers awarded to each author<br>• The full name of each funder<br>• URL of each funder website<br>• Did the sponsors or funders play any role in the study design, data collection and analysis, decision to publish, or preparation of the manuscript?<br>• **NO** - Include this sentence at the end of your statement: *The funders had no role in study design, data collection and analysis, decision to publish, or preparation of the manuscript.*<br>• **YES** - Specify the role(s) played.<br><br>* typeset	
**Competing Interests**<br><br>Use the instructions below to enter a competing interest statement for this submission. On behalf of all authors, disclose any competing interests that could be perceived to bias this work—acknowledging all financial support and any other relevant financial or non-financial competing interests.<br><br>This statement is required for submission and **will appear in the published article** if the submission is accepted. Please make sure it is accurate and that any funding sources listed in your Funding Information later in the submission form are also declared in your Financial Disclosure statement.<br><br>View published research articles from *PLOS ONE* for specific examples.	The authors have declared that no competing interests exist.

**NO authors have competing interests**<br><br>Enter: *The authors have declared that no competing interests exist.*<br><br>**Authors with competing interests**<br><br>Enter competing interest details beginning with this statement:<br><br>*I have read the journal's policy and the authors of this manuscript have the following competing interests: [insert competing interests here]*<br><br>* typeset	
**Ethics Statement**<br><br>Enter an ethics statement for this submission. This statement is required if the study involved:<br><br>• Human participants<br>• Human specimens or tissue<br>• Vertebrate animals or cephalopods<br>• Vertebrate embryos or tissues<br>• Field research<br><br>Write "N/A" if the submission does not require an ethics statement.<br><br>General guidance is provided below. Consult the submission guidelines for detailed instructions. **Make sure that all information entered here is included in the Methods section of the manuscript.**	N/A

**Format for specific study types**<br><br>**Human Subject Research (involving human participants and/or tissue)**<br>• Give the name of the institutional review board or ethics committee that approved the study<br>• Include the approval number and/or a statement indicating approval of this research<br>• Indicate the form of consent obtained (written/oral) or the reason that consent was not obtained (e.g. the data were analyzed anonymously)<br><br>**Animal Research (involving vertebrate animals, embryos or tissues)**<br>• Provide the name of the Institutional Animal Care and Use Committee (IACUC) or other relevant ethics board that reviewed the study protocol, and indicate whether they approved this research or granted a formal waiver of ethical approval<br>• Include an approval number if one was obtained<br>• If the study involved *non-human primates*, add *additional details* about animal welfare and steps taken to ameliorate suffering<br>• If anesthesia, euthanasia, or any kind of animal sacrifice is part of the study, include briefly which substances and/or methods were applied<br><br>**Field Research**<br><br>Include the following details if this study involves the collection of plant, animal, or other materials from a natural setting:<br>• Field permit number<br>• Name of the institution or relevant body that granted permission	
**Data Availability**<br><br>Authors are required to make all data underlying the findings described fully available, without restriction, and from the time of publication. PLOS allows rare exceptions to address legal and ethical concerns. See the PLOS Data Policy and FAQ for detailed information.	Yes - all data are fully available without restriction

A Data Availability Statement describing where the data can be found is required at submission. Your answers to this question constitute the Data Availability Statement and **will be published in the article**, if accepted.<br><br>**Important:** Stating 'data available on request from the author' is not sufficient. If your data are only available upon request, select 'No' for the first question and explain your exceptional situation in the text box.<br><br>Do the authors confirm that all data underlying the findings described in their manuscript are fully available without restriction?	
**Describe where the data may be found in full sentences. If you are copying our sample text, replace any instances of XXX with the appropriate details.**<br><br>• If the data are **held or will be held in a public repository**, include URLs, accession numbers or DOIs. If this information will only be available after acceptance, indicate this by ticking the box below. For example: *All XXX files are available from the XXX database (accession number(s) XXX, XXX.).*<br>• If the data are all contained **within the manuscript and/or Supporting Information files**, enter the following: *All relevant data are within the manuscript and its Supporting Information files.*<br>• If neither of these applies but you are able to provide **details of access elsewhere**, with or without limitations, please do so. For example:<br><br>*Data cannot be shared publicly because of [XXX]. Data are available from the XXX Institutional Data Access / Ethics Committee (contact via XXX) for researchers who meet the criteria for access to confidential data.*<br><br>*The data underlying the results presented in the study are available from (include the name of the third party*	Data is available upon request.

and contact information or URL).<br>• This text is appropriate if the data are owned by a third party and authors do not have permission to share the data.<br><br>* typeset	
Additional data availability information:	Tick here if your circumstances are not covered by the questions above and you need the journal's help to make your data available.

Click here to access/download;Manuscript;Manuscript.docx

Economic Freedom Index Effects on Tourist's Arrivals in European Countries: A Spatial Panel Analysis

Abstract

This paper assesses the impact of economic freedom index components on tourist arrivals in 41 European countries, the world's leading tourism destinations, between 2005 and 2018. We are the first to use a spatial panel data method and consider spatial spillover to investigate the mentioned relationship. We find an increase in neighboring countries' GDP per capita results in higher domestic tourist arrivals. Additionally, results show an increase in neighboring countries' prices increases domestic tourists' arrivals. More importantly, the economic freedom index has a significant effect on tourism arrivals in Europe. While some components of economic freedom have marginal effects, the coefficients for the Trade Freedom Index, Investment Freedom Index, Property Rights Index, and Government Integrity Index are significant and positive. Our findings have meaningful implications for policymakers interested in sustainable tourism in European countries.

Keywords**:** Spatial panel analysis; Economic freedom; European countries; Tourism

JEL: C31, N94, L83, Z32

Introduction

For an extended period, economic growth has been the primary macroeconomic objective of countries and governments that rely on the tourism industry to address chronic problems such as underemployment (López 2019). Meanwhile, numerous empirical studies have established the tourism industry's critical role in economic prosperity (Aslan 2016; Selimi, Sadiku, and Sadiku 2017; Manzoor et al. 2019; Rasool, Maqbool, and Tarique 2021). This role has been validated by theories such as the tourism-led growth theory. Thus, researchers and development theorists must understand the primary factors stimulating tourism expansion, and they have examined several demand-and-supply-side economic, political, institutional, and infrastructural factors.[1] Acemoglu, Johnson, and Robinson (2012) argue in the context of tourism-led growth theory that economic freedom components and sound institutions are critical and fundamental factors in increasing tourism demand and channeling tourism development toward economic growth. Additionally, institutional economic theory confirms that economic liberty and the quality of institutions increase the efficiency of the tourism sector.

Heritage Foundation (2020) defines economic freedom as a concept that portrays the nature of the liberal economic system, implying that individuals have the freedom to choose in a wide variety of economic activities, where all of their legal rights are assured by law. It is anticipated that free economies will foster a more competitive environment for entrepreneurs to provide higher-quality tourism goods and services (Saha, Su, and Campbell 2017). The Heritage Foundation measures this index based on four significant categories: the rule of law, market openness, regulatory efficiency, and government size. Moreover, it assumes a precise signal for investors and interpreters that it is time to start a new business. According to Ağazade (2021), low economic freedom indicators can harm tourism destinations' prospects and erode their brand values, thereby limiting tourism demand.

As many countries continue to make minimal efforts to join the global system, integrate into a globalized world, and adopt a market economic system, examining the economic freedom index's effect on tourism would be a critical area of research. Although a few studies have examined the effects of press and personal freedom, civil liberties, and economic and political freedom, corruption, and economic freedom (Su and Lin 2014; Kubickova 2016; Saha, Su, and Campbell 2017; Tang 2018; Kubickova 2019; Bulut, Kocak, and Suess 2020; Öcal and Yildirim 2010), there is a lack of comprehensive understanding on the effects of economic freedom index's components' on the tourism sector. As a result, this paper examines the impact of economic freedom index components on tourist arrivals to European countries: the world's most popular tourist destination.

On the one hand, European countries have historically benefited from tourism, with direct and indirect tourism revenues totaling 2,191 billion US dollars in 2019, accounting for approximately 10.3% of GDP and 11.7% of total employment in 2018. On the other hand, Europe is the only region with economies that enjoy relatively high levels of economic freedom, with 11 of the world's 20 most free countries located there. Most countries in the region are arranged as "mostly" and "moderately" free. Europe's average economic freedom index is 68.0, significantly higher than the global average (Miller and Kim 2017).

Furthermore, several researchers assert that the world operates as an interdependent system, in which economic advancements in one country can significantly impact neighboring countries (Lanouar and Goaied 2019; Aslan, Öcal, and Altınöz 2020; Ritchie 2004). In this context, some

[1] Some of the considered variables in the literature are: GDP, price level, currency exchange rate, travel cost, travel risk, tourism anxiety, political instability, heritage sites, cultural similarities, and corruption.

researchers have emphasized the spatial characteristics of tourism growth-related factors and confirmed that regions might benefit from beneficial spatial spillovers caused by their neighbors' thriving tourism industries (Ponce et al. 2020; Karimi, Khezri, and Razzaghi 2021; Jiao et al. 2019; Mamipour and Abdi 2017; Yang and Fik 2014). Romão and Saito (2017), for example, assume tourism as a location-oriented action with frequent interregional flows. Therefore, global and local indicators of spatial autocorrelation can help identify distinct patterns of regional tourism dynamics.

The contribution of this paper is twofold. First, this work comprehensively examines the effect of economic freedom on the tourism industry by examining the majority of the economic freedom index parameters. Second, to the best of our knowledge, this study is the first to use a spatial panel data method investigating the impacts of economic liberty on tourism. It is worth noting that conventional panel econometric frameworks frequently overlook spatial characteristics. According to Fingleton and López-Bazo (2006) and LeSage and Pace (2009), ignoring spatial spillovers in a regional tourism growth model leads to biased and potentially mendacious results. Additionally, they do not account for the economic freedom index's indirect effects (caused by neighbors) or spatial spillover effects on tourism development. As a result, spatial econometric models are more advantageous, efficient, and effective (Meng et al. 2017; You and Lv 2018).

The remainder of this paper is summarized as follows. Section 2 reviews the current literature. Section 3 describes the spatial econometrics methodology and model. Sections 4 and 5 contain the results of estimations and conclusion remarks, respectively.

Literature Review

The tourism literature has established the importance of numerous economic, political, institutional, infrastructural, and regional factors in tourism growth. However, the most frequently cited common determinants of tourism are economic factors in the destination country and its neighbors. (Lim 2006; Larsen, Brun, and Øgaard 2009; Yap and Saha 2013; Saha and Yap 2014; Cheung and Saha 2015), among others, have established a robust link between economic factors and tourism growth. For example, Saha and Yap (2014) and Rosselló, Becken, and Santana-Gallego (2020) focused on economic factors such as GDP, price level, trade openness, and economic freedom components of tourism growth in the countries mentioned above.

Forsyth and Dwyer (2009) argue that relative prices are a significant factor in tourists' destination selection processes, and they compare the price levels in the origin and destination countries. Furthermore, they compare all competing substitute destinations' price levels to determine travel costs. The relative price is countries' consumer price indexes adjusted for the mutual exchange rate. If the origin country’s currency appreciates, the destination country's tourism imports increase. The opposite also holds higher relative prices in the destination country harm tourist arrivals (Lim 1997). Moreover, according to Seetanah, Sannassee, and Rojid (2015), prices in rival destinations may affect other countries’ inbound tourists. In addition, tourists from large countries are more price-sensitive when it comes to destination selection than travelers from smaller countries with fewer options. Tourists compare living costs and choose the least expensive destination unless the competing countries are complementary rather than substitutive. Lee, Var, and Blaine (1996) identify prices and income levels as the main factors of tourism demand, and they distinguish two distinct channels through which these variables affect tourism demand: the income effect and the substitution effect. Tourists, they argue, consider the relative prices of goods and services in comparison to their direct substitutes. Conversely, Morrison (1996) states that price

level may not disincentivize tourism demand, citing the hedonistic nature of tourism-related products consumption. This suggests that there exists a positive correlation between tourism demand and price levels.

Rugg (1973), Morley (1992), and Masiero and Nicolau (2012) all propose that tourists' income levels may mitigate the effect of price levels on tourism demand. Tourists seek to maximize their utility of consumption within the constraints imposed by the cost of tourism goods and services and their budgetary/income restrictions. Then, high-income tourists can choose more expensive destinations, while low-income travelers can choose a less expensive destination. Numerous researchers consider income level as a critical determinant of tourists' decision-making. Tourists' income is an individual budget constraint that limits spending capacity. It is assumed that - when tourism is considered a normal good - middle- and high-income groups will take vacations more frequently (Hay and McConnell 1979; Walsh et al. 1992; Crawford and Godbey 1987; Davies and Mangan 1992; Middleton 1995; Mergoupis and Steuer 2003).

Gholipour, Tajaddini, and Al-mulali (2014) and Das and DiRienzo (2009) identify economic freedom as a critical determinant of tourism demand and suggest that enhancing economic freedom could boost the tourism industry's competitiveness and inbound tourism. However, the four components of the freedom index may have different effects on tourism arrivals. For example, the government size may contribute to improving touristic infrastructures. Moreover, business freedom and tax burden and might be related to the extensive use of entrepreneurial activities, and entrepreneurs would provide a range of qualified and competitive tourist services. In this context, Easton and Walker (1997), Knack and Keefer (1995), and Barro and Sala-i-Martin (2004) confirm that free nations are establishing efficient labor market and product market, a stable monetary and legal system, and open trade that make the business environment more competitive.

Furthermore, trade freedom is critical, as it contributes to the diversity of goods and services, thereby increasing visitor satisfaction. Moreover, countries suffering from limited economic freedom may encounter tourists with an erratic social system, an ineffective judiciary, and a corrupt economic system, causing problems for tourists and possibly landing them in trouble (Saha, Su, and Campbell 2017). The developed competition conditions created by a destination country's economic freedom encourage tourism service providers to offer more qualified tourism goods and services at more competitive prices, piquing tourists' interest in visiting the destination country.

There is a remarkable body of literature assessing the nexus between economic freedom components and the tourism industry by examining the relationship between tourism sector development and various facets of the legal system, including property rights, civil rights, contract enforcement, and infrastructure investments. Legal systems and institutions must safeguard all individuals' civil rights and property against aggressive behavior. Contracts must be adequately enforced, effectively, and expeditiously by legal institutions. Indeed, institutions contribute to an increase in the efficiency of nearly every economic sector. As a result, a significant positive effect on international tourism can be assumed (Acemoglu, Johnson, and Robinson 2012). Ozcan, Aslan, and Nazlioglu (2017) suggest that political economy encompasses numerous subfields, including institutional economics.

Infrastructure investments required for tourism development are typically long-term and costly. Thus, privately held companies that make these investments may be necessary to resolve potential conflicts through a practical and integrated official system rather than a constantly changing political authority. As a result, the legal system's effectiveness benefits the tourism sector by diminishing corruption. Notably, foreign investors consider the legal system's efficacy and

corruption level when deciding which country's tourism sector to finance (Saha, Su, and Campbell 2017).

Meanwhile, several studies examine the influence of economic freedom indices on tourism development without considering neighboring countries' spatial dependencies. For example, Knack and Keefer (1995) and Acemoglu, Johnson, and Robinson (2012) conducted empirical analyses to determine whether economic freedom impacts the tourism industry's ability to grow economically without considering spillover effects. Their findings indicate that economic freedom impacts tourism arrivals positively. Saha and Yap ((2014) investigated the impact of terrorism and political unreliability on tourism in 139 countries from 1999 to 2009. The authors found that political unreliability and terrorism impact tourism development adversely. Moreover, Balli, Balli, and Jean Louis (2016) showed that institutional quality, used as a proxy for economic freedom, is the primary factor influencing tourism destination selection in 34 of the 38 OECD countries.

Ozcan, Aslan, and Nazlioglu (2017) investigated the nexus between foreign tourist arrivals and economic liberty in 17 post-socialist transition countries from 1996 to 2012. The authors demonstrate the adequacy of economic freedom and growth and the adequacy of economic growth and international tourism. They found that only economic freedom stimulates tourism in 16 of the 17 post-socialist transition countries.

Samitas et al. (2018) found that the civil liberties index promotes inbound tourism. Gozgor et al. (2019) demonstrated that an efficient legal system and enhanced property rights protection encourage tourism. According to Kubickova (2019), labor freedom, monetary freedom, and business freedom significantly impact tourism in seven Central American countries, while freedom from corruption and property rights have a negligible effect on destination competitiveness. Aslan, Öcal, and Altınöz (2020) showed a bidirectional causality between tourist arrivals and indicators of economic freedom; they found that a rise in economic freedom indices affected tourist arrivals in Mediterranean countries adversely. Bulut, Kocak, and Suess (2020) demonstrated that the degree of freedom might help explain the volume of tourist arrivals in eight of the most visited countries in the world. None of these studies summarized above consider the inter-and intra-dynamics effects of tourism on the neighboring countries. This paper fills this significant gap in the current literature by using a spatial econometrics approach.

Methodology

The tourism arrival is the dependent variable in the current study, which is a function of the logarithms of GDP per capita ($lnGDP$), the logarithm of destination country prices ($lnPRICE$), and the logarithm of trade openness ($lnOPE$). Equation 1 summarizes this function. The variables are transformed into logarithmic form; therefore, corresponding coefficients are elasticities. To take the destination country's size into account, tourist arrivals data is divided by population and expressed in terms of per capita. The logarithm of real GDP per capita was used as a proxy for each destination's development level (Saha and Yap 2014; Rosselló, Becken, and Santana-Gallego 2020).

$$lnTOUR_{it} = \beta_1 + \beta_2 lnGDP_{it} + \beta_3 lnPRICE_{2it} + \beta_4 lnOPE_{it} + c_i(optional) + \alpha_t(optional) + \upsilon_{it} \quad (1)$$

The objective here is to determine the regional effects of our independent variables on country-level tourist arrivals. Due to the spatial nature of the effects of neighboring country variables on domestic arrivals, following Tian et al. (2021), we used three spatial panel data models to examine

such spillovers. That includes: 1) a lagged dependent variable model, 2) a model with the spatial autoregressive process in the disturbance term, and 3) a spatial Durbin model, which incorporates the effects of a neighboring country's exogenous variables on the dependent variable in the origin country. The spatial lag model is formulated as follows:

$$y_{it} = \lambda \sum_{j=1}^{N} w_{ij} y_{jt} + \varphi + \beta x_{it} + c_i(optional) + \alpha_t(optional) + \upsilon_{it} \tag{2}$$

Where y_{it} represents the dependent variable, and x_{it} is a 1×K vector of exogenous variables for countries $i = 1, \dots, N$ at periods $t = 1, \dots, T$. $\sum_{j=1}^{N} w_{ij} y_{jt}$ captures the impact of the dependent variables y_{jt} in neighboring countries on the dependent variable y_{it} in a destination country, and λ denotes the corresponding coefficient. w_{ij} is the $i, j - th$ element of w, where w is the spatial weights matrix. Prior to matrix standardization, the $i, j - th$ element will have the value of one of two neighboring countries or zero if they are not neighbors. c_i are country-specific intercepts that capture heterogeneity across countries, while α_t are period-specific intercepts that capture heterogeneity across periods. The omission of the latter two variables might bias the estimates in a common cross-sectional and time-series study, respectively (Baltagi 2005). υ_{it} is the random disturbance term.

The spatial error model consists of a disturbance term for country i, u_{it}, which depends on the disturbance terms of adjacent countries j, u_{jt}, w, and an idiosyncratic term υ_{it}:

$$\begin{gathered} y_{it} = \lambda \sum_{j=1}^{N} w_{ij} y_{jt} + \varphi + x_{it}\beta + c_i(optional) + \alpha_t(optional) + u_{it} \\ u_{it} = \rho \sum_{j=1}^{N} w_{ij} u_{jt} + \upsilon_{it} \end{gathered} \tag{3}$$

Finally, the spatial Durbin model incorporates spatial lagged exogenous variables into the spatial lag model as follows:

$$y_{it} = \lambda \sum_{j=1}^{N} w_{ij} y_{jt} + \varphi + x_{it}\beta + \sum_{j=1}^{N} w_{ij} x_{jt}\, \theta + c_i(optional) + \alpha_t(optional) + \upsilon_{it} \tag{4}$$

Where $\sum_{j=1}^{N} w_{ij} x_{jt}$ captures the interaction term of exogenous variables x_{ijt} in adjacent countries on the dependent variable y_{it} in a particular country. Additionally, θ denotes a $K \times 1$ vector of parameters.

Data

Tables 1 and 2 summarize, respectively, the variables used in the analyses and descriptive statistics for the data. We collected and used data from 41 European countries from 2005 to 2018 to model the spatial effects of tourism arrivals determinants. Figure 1 illustrates the possible spatial relationship between two variables by visualizing tourist arrivals per capita and the total economic freedom index in our sample. A commonly used test, namely "Moran's I," was employed to examine such spillover effects thoroughly. The computed statistic reveals the spatial effects depicted in Figure 2. Two dimensions of Figure 2 are the regional observations of variables and the associated spatial lag data. Positive Moran's I values in Quadrants I and III confirm the spatial accumulation of similar values throughout the region; while a negative Moran's I can be configured in Quadrants II and IV. According to the fitting lines, positive autocorrelation dominates for most countries, indicating that neighboring countries have comparable tourist arrivals and economic

freedom indexes, particularly for the latter. As a result, spatial econometric models must be utilized to assess the impacts of determinants of tourist arrivals.

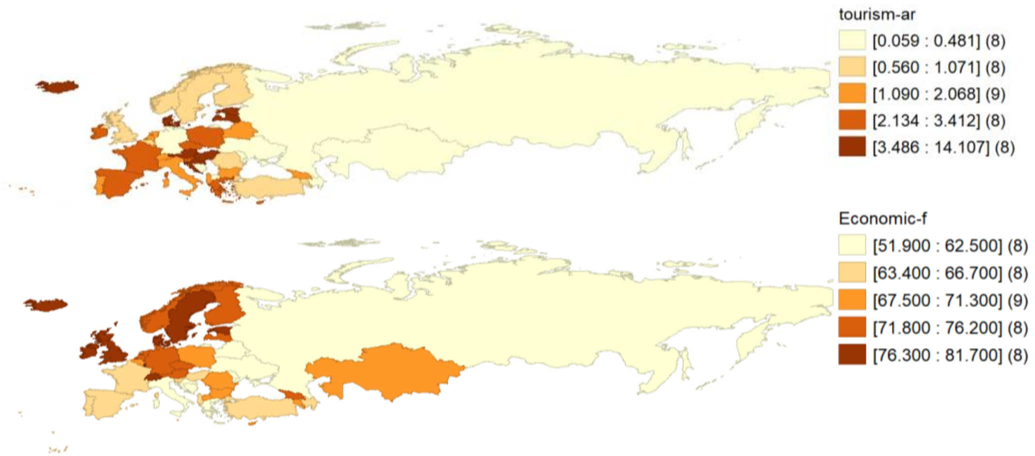

Fig. 1. Tourism arrivals per capita & total economic freedom index

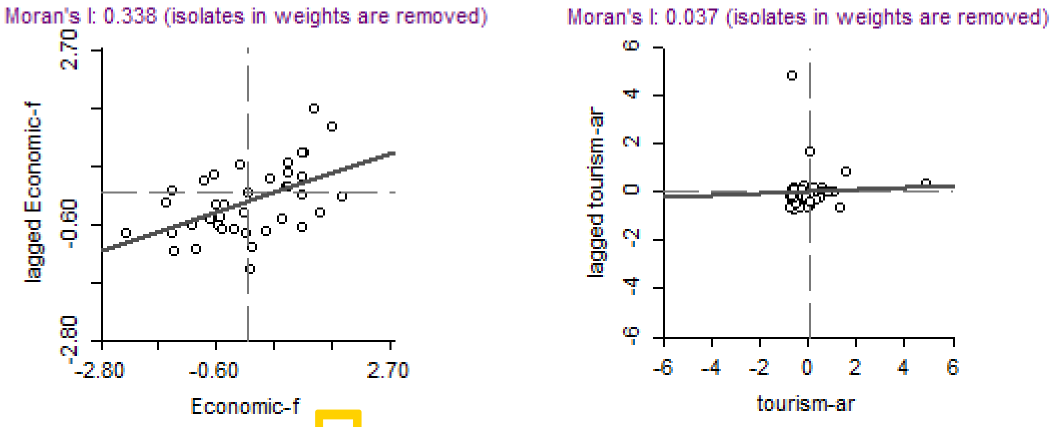

Fig. 2. Moran's I index across assessed European countries

Table 1: Variables definitions and notations

Variable used	Variable structure	Source
$lnTOUR_{it}$	$lnTOURISM_{it} = log(TOURISM_{it})$<br>$TOURISM_{it}$= Tourist arrival per capita	UNWTO
$lnGDPP_{it}$	$lnGDPP_{it} = log(GDPP_{it})$<br>$GDPP_{it}$= GDP per capita (2010 USD)	WDI
$lnPRICE_{it}$	$lnPRICE_{it} = \log(PRICE_{it})$<br>$PRICE_{it}$= PPP conversion factor divided by market exchange rate	WDI
$lnOPE_{it}$	$lnOPE_{it} = \log(OPE_{it})$<br>$OPE_{it}$= Trade Openness (as a percentage of GDP)	WDI
$lnEFI_{it}$	$lnEFI_{it} = log(EFI_{it})$<br>$EFI_{it}$= Total Economic Freedom Index	Heritage
$lnBFI_{it}$	$lnBFI_{it} = log(BFI_{it})$<br>$BFI_{it}$= Business Freedom Index	Heritage
$lnLFI_{it}$	$lnLFI_{it} = log(LFI_{it})$	Heritage

	LFI_{it}= Labor Freedom Index	
$lnLMF_{it}$	$lnLMF_{it} = log(LMF_{it})$<br>$LMF_{it}$= Monetary Freedom Index	Heritage
$lnTFI_{it}$	$lnTFI_{it} = log(TFI_{it})$<br>$TFI_{it}$= Trade Freedom Index	Heritage
$lnIFI_{it}$	$lnIFI_{it} = log(IFI_{it})$<br>$IFI_{it}$= Investment Freedom Index	Heritage
$lnFFI_{it}$	$lnFFI_{it} = log(FFI_{it})$<br>$FFI_{it}$= Financial Freedom Index	Heritage
$lnPRI_{it}$	$lnPRI_{it} = log(PRI_{it})$<br>$PRI_{it}$= Property Rights Index	Heritage
$lnGII_{it}$	$lnGII_{it} = log(GII_{it})$<br>$GII_{it}$= Government Integrity Index	Heritage
$lnTBI_{it}$	$lnTBI_{it} = log(TBI_{it})$<br>$TBI_{it}$= Tax Burden Index	Heritage
$lnGSI_{it}$	$lnGSI_{it} = log(GSI_{it})$<br>$GSI_{it}$= Government Spending Index	Heritage

Table 2

Summary statistics of key variables from 2005 to 2018

Variable	Mean	Median	Maximum	Minimum	Std. Dev.	Observations
$lnTOUR_{it}$	13.776	13.916	16.480	9.950	1.197	574
$lnGDPP_{it}$	9.817	9.993	11.626	7.631	1.030	574
$lnPRICE_{it}$	-0.365	-0.335	0.518	-1.628	0.478	574
$lnOPE_{it}$	4.574	4.517	6.012	3.816	0.435	574
$lnEFI_{it}$	4.189	4.207	4.414	3.807	0.127	574
$lnBFI_{it}$	4.314	4.332	4.605	3.656	0.171	574
$lnLFI_{it}$	4.096	4.101	4.605	3.434	0.234	574
$lnLMF_{it}$	4.361	4.383	4.519	3.523	0.105	574
$lnTFI_{it}$	4.426	4.459	4.500	3.789	0.077	574
$lnIFI_{it}$	4.161	4.248	4.554	2.303	0.379	574
$lnFFI_{it}$	4.099	4.094	4.500	2.303	0.361	574
$lnPRI_{it}$	4.025	4.243	4.554	2.303	0.494	574
$lnGII_{it}$	3.933	3.970	4.575	2.890	0.449	574
$lnTBI_{it}$	4.224	4.273	4.544	3.487	0.238	574
$lnGSI_{it}$	3.595	3.780	4.510	-0.693	0.811	574

Due to the short duration of some index dimensions, our empirical model only includes the following indexes: 1) *Business Freedom:* defined as people's ability to start a business without government interposition. 2) *Labor Freedom:* defined as an individual's ability to find work and a business's ability to contract for labor and release unneeded workers freely. 3) *Monetary Freedom:* defined as the product of the price stability and price control criteria. 4) *Trade Freedom:* defined as the removal of every tariff and non-tariff barrier that imposes restrictions on international trade. 5) *Investment Freedom:* defined as the absence of restrictions on capital investment streams, allowing firms and individuals to allocate resources between activities freely. 6) *Financial Freedom:* defined as the degree to which individuals are supported in making life choices without being unduly constrained by financial constraints. 7) *Property Rights:* defined as the private sector's ability to accumulate wealth and property freely. 8) *Government Integrity:* defined as practices that protect government institutions from systemic corruption. 9) *Tax Burden:* defined as

every kind of direct and indirect taxes. 10) *Government Spending:* defined as the total amount of money spent by the government.

The three main sources of our data include the World Development Indicator (WDI), the United Nations World Tourism Organization (UNWTO), and The Heritage Foundation (Heritage).[2]

Results

Initially, we conducted several diagnostic tests to establish the optimal model. The existence of fixed effects in the model is examined in this study using different likelihood ratio (LR) tests in Table 3. If the null hypothesis can be rejected, a fixed-effects model that includes spatial and time-period terms simultaneously will be the suitable model for the empirical analyses. If the opposite holds, the time-period effects or spatial effects, depending on which one is the base model, will be the appropriate model. The test results provide evidence to reject the null hypotheses in all models. Therefore, we can conclude that a fixed-effects model that includes spatial and time-period terms simultaneously is the optimal model for the empirical analyses based on this exercise.

Table 3
The Likelihood Ratio test results

	Spatial effects		Time-period effects	
Model 1	80.411	(0.000)	1875.501	(0.000)
Model 2	83.087	(0.000)	1882.232	(0.000)
Model 3	78.761	(0.000)	1869.351	(0.000)
Model 4	81.846	(0.000)	1874.402	(0.000)
Model 5	79.682	(0.000)	1872.735	(0.000)
Model 6	71.935	(0.000)	1872.274	(0.000)
Model 7	66.086	(0.000)	1865.733	(0.000)
Model 8	81.562	(0.000)	1874.699	(0.000)
Model 9	86.304	(0.000)	1899.817	(0.000)
Model 10	87.351	(0.000)	1897.743	(0.000)
Model 11	84.725	(0.000)	1878.683	(0.000)
Model 12	79.195	(0.000)	1873.057	(0.000)

Note: The values in parentheses indicate p-values.
Source: Authors' estimations.

To determine the presence of spatial interaction effects when the spatial error or spatial lag is included, we use the Lagrange-Multiplier (LM) test summarized in Table 4. If the null hypothesis can be rejected, we can confirm the existence of both the spatial lagged model and the spatial error model. For all models that include time and spatial effects simultaneously, we can reject the null hypotheses. Therefore, these test results offer enough evidence that our empirical model should contain the spatial interaction effects.

[2] Online access to the data is available at https://www.unwto.org/data, www.heritage.org/index, and www.datacatalog.worldbank.org/dataset/world-development-indicators.

Table 4
The LM test is used to determine whether models contain spatial lag or spatial error.

		Spatial effects		Time-period effects		Spatial and time-period effects	
Model 1	LM spatial lag	20.252	(0.000)	2.370	(0.124)	8.300	(0.004)
	LM spatial error	10.991	(0.001)	0.078	(0.780)	7.878	(0.005)
Model 2	LM spatial lag	19.192	(0.000)	2.758	(0.097)	8.091	(0.004)
	LM spatial error	8.987	(0.003)	0.162	(0.687)	5.678	(0.017)
Model 3	LM spatial lag	16.097	(0.000)	5.959	(0.015)	6.730	(0.009)
	LM spatial error	8.175	(0.004)	0.906	(0.341)	5.778	(0.016)
Model 4	LM spatial lag	20.368	(0.000)	1.458	(0.227)	7.709	(0.005)
	LM spatial error	11.398	(0.001)	0.007	(0.936)	6.673	(0.010)
Model 5	LM spatial lag	19.579	(0.000)	2.864	(0.091)	8.308	(0.004)
	LM spatial error	10.321	(0.001)	0.182	(0.669)	7.893	(0.005)
Model 6	LM spatial lag	15.024	(0.000)	3.333	(0.068)	7.899	(0.005)
	LM spatial error	6.856	(0.009)	0.310	(0.578)	6.340	(0.012)
Model 7	LM spatial lag	16.203	(0.000)	3.244	(0.072)	7.804	(0.005)
	LM spatial error	5.754	(0.016)	0.288	(0.592)	5.656	(0.017)
Model 8	LM spatial lag	20.042	(0.000)	2.123	(0.145)	7.941	(0.005)
	LM spatial error	10.669	(0.001)	0.002	(0.968)	7.097	(0.008)
Model 9	LM spatial lag	20.122	(0.000)	1.272	(0.259)	9.517	(0.002)
	LM spatial error	12.441	(0.000)	0.071	(0.790)	7.249	(0.007)
Model 10	LM spatial lag	17.634	(0.000)	2.399	(0.121)	6.144	(0.013)
	LM spatial error	8.906	(0.003)	0.087	(0.768)	4.406	(0.036)
Model 11	LM spatial lag	20.515	(0.000)	2.320	(0.128)	7.453	(0.006)
	LM spatial error	11.000	(0.001)	0.210	(0.647)	7.119	(0.008)
Model 12	LM spatial lag	18.836	(0.000)	2.712	(0.100)	8.586	(0.003)
	LM spatial error	10.264	(0.001)	0.074	(0.786)	8.021	(0.005)

Note: The values in parentheses indicate p-values.
Source: Authors' estimations.

To test whether the fixed-effect models should be replaced with random-effects, we use the Hausman test. Hausman's test null hypothesis confirms the existence of random effects. Test results are summarized in Table 5. We reject the null hypotheses at a 1% significance level in the majority of the tests indicating that fixed effects are confirmed in the models.

Table 5
Hausman test results

	spatial lag model		spatial Durbin model	
Model 1	86.787	(0.000)	41.850	(0.000)
Model 2	177.240	(0.000)	0.776	(1.000)
Model 3	64.670	(0.000)	36.094	(0.000)
Model 4	8.790	(0.118)	53.537	(0.000)
Model 5	201.902	(0.000)	34.723	(0.000)
Model 6	196.124	(0.000)	46.395	(0.000)

Model 7	62.232	(0.000)	23.744	(0.005)
Model 8	41.187	(0.000)	30.083	(0.000)
Model 9	81.513	(0.000)	43.392	(0.000)
Model 10	74.860	(0.000)	28.858	(0.001)
Model 11	104.451	(0.000)	34.572	(0.000)
Model 12	78.027	(0.000)	42.087	(0.000)

Note: The values in parentheses indicate p-values.
Source: Authors' estimations.

Furthermore, we test the following two null hypotheses $H_0: \theta = 0$ and $H_0: \theta + \lambda\beta = 0$ using Equation (4) to determine whether the Durbin model can be converted into the lag model and the error model, respectively. In so doing, we employ the LR and Wald tests. Table 6 summarized these tests results. The Wald test values are significant, indicating that the Durbin model cannot be converted to an error or lag model in all cases. Hence, we utilize a spatial Durbin model with spatially lagged independent variables for the empirical analyses.

Table 6
A spatial Durbin model compared to a spatial error and lag model.

	Wald test				LR test			
	spatial Durbin model against lag model		spatial Durbin model against error model		spatial Durbin model against lag model		spatial Durbin model against error model	
Model 1	7.506	(0.057)	19.254	(0.000)	8.106	(0.044)	20.872	(0.000)
Model 2	14.398	(0.006)	26.847	(0.000)	15.590	(0.004)	29.111	(0.000)
Model 3	13.010	(0.011)	23.596	(0.000)	14.019	(0.007)	25.327	(0.000)
Model 4	10.626	(0.031)	21.694	(0.000)	11.395	(0.022)	23.542	(0.000)
Model 5	12.907	(0.012)	24.673	(0.000)	13.884	(0.008)	26.624	(0.000)
Model 6	10.107	(0.039)	20.945	(0.000)	10.947	(0.027)	22.410	(0.000)
Model 7	37.988	(0.000)	49.624	(0.000)	39.999	(0.000)	53.145	(0.000)
Model 8	8.400	(0.078)	20.095	(0.000)	9.050	(0.060)	21.795	(0.000)
Model 9	5.581	(0.233)	15.832	(0.003)	5.997	(0.199)	17.052	(0.002)
Model 10	10.755	(0.029)	22.349	(0.000)	11.630	(0.020)	23.691	(0.000)
Model 11	7.657	(0.105)	19.852	(0.001)	8.307	(0.081)	21.431	(0.000)
Model 12	9.187	(0.057)	20.141	(0.000)	9.876	(0.043)	21.907	(0.000)

Note: The values in parentheses indicate p-values.
Source: Authors' estimations.

Tables (7) and (8) illustrate the estimation results for various models. To avoid the collinearity issue among various indicators of economic freedom, we entered these variables into distinct, independent models. All models demonstrate that common control variables significantly affect tourist arrivals. Each percentage point increase in GDP per capita yields 1.5 percentage points increase in tourist arrivals. Positive and significant effects on trade openness are also established. Each percentage point increase in trade openness results in an approximately 0.54 percent increase in tourist arrivals.

Although marginally significant, the increase in destination country prices positively affects tourist arrivals. This theoretical contradiction is highlighted when the coefficient of variation of neighboring countries' prices is considered ($W \times lnPRICE$). According to the variable coefficient, each percent increase in the price level results in a decrease of approximately 0.3% in the number of tourists entering the country. As per theoretical predictions, an increase in the price level should increase the prices of goods and services in the destination countries, thereby enhancing the destination country's competitiveness. Naturally, the expectations emphasize the price effects of tourist demand. A review of Figure 3 reveals that the price levels and tourist arrivals had a positive correlation for 170 countries in 2017.

Due to Europe's open economic structure, the difference in the general level of prices among countries primarily stems from disparities in the prices of non-tradable goods and services. The price level in the service sector, in particular, is the primary factor determining the difference in price levels. Increased service sector prices may indicate that countries have a higher GDP per capita. As a result, infrastructure development and welfare can be expected to be more inclusive in countries with higher prices. As a result, European countries experience a greater prevalence of price effects on the supply of tourism services. Even an increase in neighboring countries' prices results in a flow of tourists to neighboring countries and a decline in domestic entries.

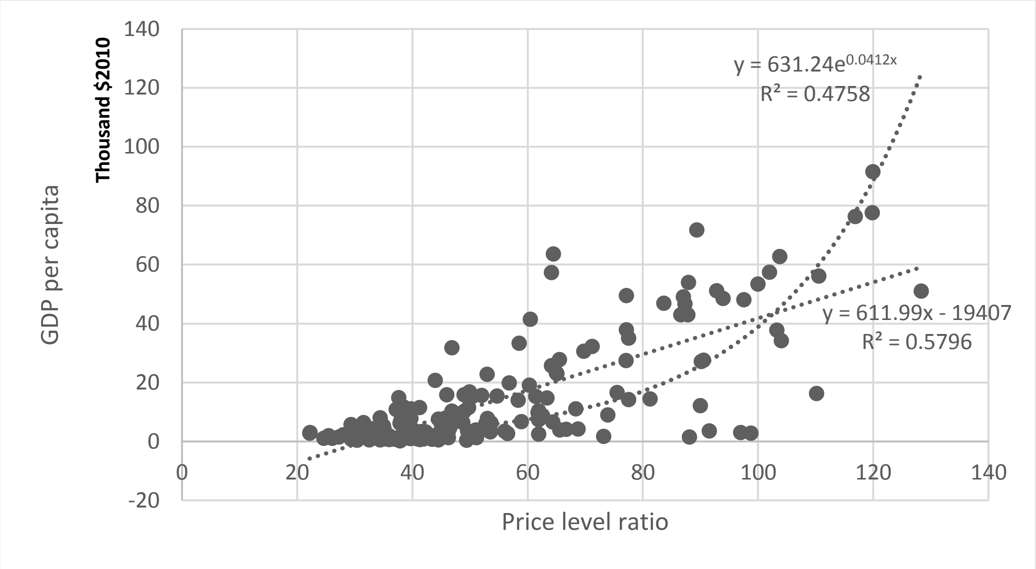

Fig. 3. Positive correlation between tourist arrivals per capita & level of prices (Source: WDI)

In Model 2, the total economic freedom variable coefficient is positively marginally significant. Examining the effects of its constituents reveals that the source of such meaningless effects is the conflicting influences between its constituents. While the coefficients of six economic freedom indices are insignificant, the Trade Freedom Index, the Investment Freedom Index, the Property Rights Index, and the Government Integrity Index are all significant and positive.

Table 7
Models 1 to 6: estimation results

	Model 1	Model 2	Model 3	Model 4	Model 5	Model 6
$lnGDPP$	1.533	1.435	1.458	1.540	1.514	1.370
	(0.000)	(0.000)	(0.000)	(0.000)	(0.000)	(0.000)
$lnPRICE$	0.185	0.200	0.200	0.188	0.211	0.195
	(0.151)	(0.118)	(0.120)	(0.145)	(0.102)	(0.124)
$lnOPE$	0.531	0.552	0.577	0.541	0.539	0.506
	(0.000)	(0.000)	(0.000)	(0.000)	(0.000)	(0.000)
$lnEFI$		0.286				
		(0.279)				
$lnBFI$			0.131			
			(0.270)			
$lnLFI$				-0.012		
				(0.905)		
$lnMFI$					-0.027	
					(0.855)	
$lnTFI$						0.729
						(0.000)
$W \times lnGDPP$	-0.020	-0.216	-0.164	-0.051	0.004	-0.124
	(0.909)	(0.255)	(0.373)	(0.776)	(0.984)	(0.492)
$W \times lnPRICE$	-0.356	-0.331	-0.392	-0.380	-0.399	-0.388
	(0.011)	(0.018)	(0.005)	(0.007)	(0.004)	(0.005)
$W \times lnOPE$	-0.025	0.028	-0.007	-0.032	-0.007	-0.061
	(0.888)	(0.871)	(0.967)	(0.854)	(0.967)	(0.724)
$W \times lnEFI$		0.957				
		(0.012)				
$W \times lnBFI$			0.341			
			(0.044)			
$W \times lnLFI$				0.334		
				(0.073)		
$W \times lnMFI$					-0.601	
					(0.021)	
$W \times lnTFI$						0.231
						(0.287)
$W \times lnTOUR$	0.228	0.220	0.219	0.225	0.236	0.202
	(0.000)	(0.000)	(0.000)	(0.000)	(0.000)	(0.000)

Note: The values in parentheses indicate p-values.
Source: Authors' estimations.

Table 8
Models 7 to 12: estimation results

	Model 7	Model 8	Model 9	Model 10	Model 11	Model 12
$lnGDPP$	1.355	1.549	1.459	1.403	1.541	1.523
	(0.000)	(0.000)	(0.000)	(0.000)	(0.000)	(0.000)
$lnPRICE$	0.203	0.180	0.166	0.188	0.180	0.193
	(0.104)	(0.163)	(0.192)	(0.141)	(0.163)	(0.136)
$lnOPE$	0.541	0.509	0.472	0.554	0.517	0.531
	(0.000)	(0.000)	(0.000)	(0.000)	(0.000)	(0.000)
$lnIFI$	0.197					
	(0.003)					
$lnFFI$		-0.020				
		(0.789)				
$lnPRI$			0.251			
			(0.000)			
$lnGII$				0.214		
				(0.006)		
$lnTBI$					-0.144	
					(0.468)	
$lnGSI$						-0.004
						(0.851)
$W \times lnGDPP$	-0.228	-0.036	-0.002	-0.201	-0.025	0.008
	(0.198)	(0.841)	(0.989)	(0.273)	(0.889)	(0.965)
$W \times lnPRICE$	-0.255	-0.354	-0.296	-0.287	-0.353	-0.331
	(0.061)	(0.011)	(0.032)	(0.039)	(0.012)	(0.019)
$W \times lnOPE$	-0.026	0.004	-0.026	0.105	-0.015	-0.057
	(0.878)	(0.983)	(0.880)	(0.551)	(0.932)	(0.749)
$W \times lnIFI$	0.648					
	(0.000)					
$W \times lnFFI$		0.116				
		(0.349)				
$W \times lnPRI$			-0.064			
			(0.486)			
$W \times lnGII$				0.220		
				(0.072)		
$W \times lnTBI$					0.100	
					(0.731)	
$W \times lnGSI$						-0.048
						(0.176)
$W \times lnTOUR$	0.159	0.227	0.230	0.221	0.233	0.220
	(0.001)	(0.000)	(0.000)	(0.000)	(0.000)	(0.000)

Note: The values in parentheses indicate p-values.
Source: Authors' estimations.

Spatial models permit the separation of variables' direct and spillover effects. Direct effects quantify the impact of exogenous variables on the country's tourism. In contrast, spillover effects quantify the impact of exogenous variables in neighboring countries on a specific country's tourism. The spillover and direct effects of the common variables in Model 1 and the economic freedom indices in Models 2-12 are summarized in Table 9.

Table 9
Tourism determinants' marginal effects

	Direct		Indirect		Total	
	Coefficient	p-value	Coefficient	p-value	Coefficient	p-value
$lnGDPP$	1.559	(0.000)	0.408	(0.025)	1.967	(0.000)
$lnPRICE$	0.162	(0.194)	-0.390	(0.007)	-0.228	(0.021)
$lnOPE$	0.542	(0.000)	0.100	(0.613)	0.642	(0.003)
$lnEFI$	0.380	(0.156)	1.253	(0.007)	1.633	(0.003)
$lnBFI$	0.152	(0.198)	0.450	(0.032)	0.601	(0.008)
$lnLFI$	0.012	(0.911)	0.401	(0.092)	0.412	(0.158)
$lnMFI$	-0.066	(0.674)	-0.747	(0.022)	-0.813	(0.038)
$lnTFI$	0.756	(0.000)	0.448	(0.076)	1.205	(0.000)
$lnIFI$	0.233	(0.001)	0.774	(0.000)	1.007	(0.000)
$lnFFI$	-0.011	(0.878)	0.130	(0.400)	0.118	(0.503)
$lnPRI$	0.251	(0.000)	-0.008	(0.936)	0.243	(0.017)
$lnGII$	0.233	(0.004)	0.325	(0.035)	0.558	(0.002)
$lnTBI$	-0.146	(0.460)	0.069	(0.845)	-0.077	(0.852)
$lnGSI$	-0.007	(0.719)	-0.056	(0.200)	-0.063	(0.233)

Note: The values in parentheses indicate p-values.
Source: Authors' estimations.

According to the results summarized in Table 9, each percent increase in neighboring countries' GDP per capita results in an increase of approximately 0.4 percent in domestic tourist arrivals. This increase could be attributed to the arrival of tourists from neighboring countries, regional tourism industry development, or the overall increase in tourists in the developed world. Additionally, as previously stated, an increase in neighboring countries' prices will increase inbound domestic tourists. The neighboring countries' total economic freedom index demonstrates positive and significant effects. Furthermore, most economic freedom variables, such as the Business Freedom Index, the Investment Freedom Index, the Labor Freedom Index, the Trade Freedom Index, and the Government Integrity Index, exhibit a statistically significant and positive effect. However, neighboring countries' Monetary Freedom Index results in a decrease in the number of domestic arrivals. Among the various indicators, only improvements in the country's and neighboring countries' Investment Freedom Index and Government Integrity Index increase domestic arrivals. However, the Financial Freedom Index, the Tax Burden Index, and the Government Spending Index demonstrate no significant effect.

Discussions

According to tourism-led growth models, economic freedom and its components are expected to be crucial determinants in the tourism industry and boost tourism arrivals by generating positive externalities. Using these theories, the primary objective of this research was to examine the economic freedom spillover effects of neighboring countries on tourist arrivals in European countries. Moreover, this study examined the spatial interaction between these countries' tourism industries and the spatial movement of economic freedom within the region. Diagnostic test analyses confirmed the spatial characteristics of regional tourism growth and the spillover effects of economic freedom on European tourism arrivals. This finding is consistent with Karimi, Khezri, and Razzaghi (2021), who found evidence of regional spatial interaction in the tourism industry. The spatial Durbin model was then chosen as the best spatial econometric model for studying the factors that influence regional tourism arrivals.

While the effects of some variables corroborated theoretical predictions, the effects of others revealed disparate realities in the region. According to the findings, each percent increase in neighboring countries' GDP per capita results in an approximately 0.4 percent increase in domestic tourist arrivals. In other words, as a result of neighboring countries' developed tourism industries, the domestic country has seen an increase in tourist arrivals. This finding is consistent with the theory and with the findings of (Del P. Pablo-Romero and Molina 2013; Saha and Yap 2014; Ghalia et al. 2019; Karimi, Khezri, and Razzaghi 2021). Economic growth accelerates the development of tourist infrastructure in the country of origin. Additionally, economic growth in neighboring countries may increase international demand for the origin country's tourism (because of increased income levels in neighboring countries). Moreover, as neighboring countries develop their tourism infrastructure due to economic growth, tourists from other countries can travel to neighboring countries with more developed tourism infrastructure.

Furthermore, the results indicate that an increase in neighboring countries' prices increased inbound domestic tourists. The results of Masiero and Nicolau (2012) and Kang et al. (2018) corroborate this finding. Also, we find trade liberalization has a considerable impact on tourism arrivals. This finding is consistent with those of (Chaisumpunsakul and Pholphirul 2018; Suresh et al. 2018; Habibi and Ahmadzadeh 2015; Kulendran and Wilson 2000; Shan and Wilson 2001).

One of the paper's central findings is that the total economic freedom index has a meaningless and low-level positive significant effect on tourism arrivals in the European region. This finding is consistent with that of Aslan, Öcal, and Altınöz (2020). The European region is made up of numerous nations that experience varying degrees of economic freedom. Switzerland, for example, is one of the world's five truly "free" economies; Ukraine, Russia, and Belarus, among other eight nations, are classified as repressive and primarily unfree. Poland, Italy, France, and Spain, among the other 17 nations, have moderately free economies, while only 18 of 45 European countries have economies classified as "mostly free". In addition, these countries are struggling with a variety of policy impediments, including costly labor regulations and excessively protective, various market-distorting subsidies, increased tax burdens, and persistent fiscal problems resulting from public-sector expansion. As a result, economic growth has been stagnant, exacerbating the burden of fiscal deficits and mounting debt in several countries throughout the region. Taken together, it is acceptable that the economic freedom index has a negligible effect on tourism arrivals in Europe. Furthermore, while the coefficients for six economic freedom indices are meaningless, the coefficients for the Trade Freedom Index, the Investment Freedom Index, the Property Rights Index, and the Government Integrity Index are significantly positive. According

to 2018 Heritage Foundation reports, these findings are significant because the European region's average scores on judicial effectiveness, property rights, investment freedom, financial freedom, and government integrity outperform the global averages.

Moreover, the effect of specific components of the economic freedom index from neighboring countries on the arrival of tourists in the domestic country is remarkable. Most of the economic freedom indicators, including the Business Freedom, Trade Freedom, Labor Freedom, Investment Freedom, and Government Integrity indices, indicate that each country has a substantial positive influence, and only improvements in neighboring countries' Investment Freedom and Government Integrity measures will benefit the domestic tourism industry. According to Kırant Yozcu (2017), the effect of each of these components confirms the previously mentioned positive relationship but does not consider the effect of these factors in neighboring countries. Furthermore, contrary to Aslan, Öcal, and Altınöz (2020), we find no evidence that the Financial Freedom, Government Spending, and Tax Burden indices significantly impact the tourism industry in the sample countries. This is foreseeable in light of our sample's financial development levels.

Our findings provide a meaningful contribution to the ongoing tourism literature. We are arguably the first to consider the spatial impacts of tourism in European countries. Our results suggest that policymakers must consider the beneficial effect of investment freedom and government integrity in neighboring countries to enact an overarching and effective policy. Lastly, future research should examine the threshold values for the tourism determinants studied in this paper.

References

Acemoglu, Daron, Simon Johnson, and James A. Robinson. 2012. “The Colonial Origins of Comparative Development: An Empirical Investigation: Reply.” *American Economic Review* 102 (6): 3077–3110. https://doi.org/10.1257/aer.102.6.3077.

Ağazade, Seymur. 2021. “Institutional Quality and Tourism: Panel Causality Analysis in the Case of Mediterranean Countries.” *Almatourism-Journal of Tourism, Culture and Territorial Development* 12 (23): 41–58.

Anselin, Luc, Julie Le Gallo, and Hubert Jayet. 2008. “Spatial Panel Econometrics.” In *Advanced Studies in Theoretical and Applied Econometrics*, 46:625–60. Springer. https://doi.org/10.1007/978-3-540-75892-1_19.

Aslan, Alper. 2016. “Does Tourism Cause Growth? Evidence from Turkey.” *Current Issues in Tourism* 19 (12): 1176–84. https://doi.org/10.1080/13683500.2015.1015970.

Aslan, Alper, Oğuz Öcal, and Buket Altınöz. 2020. “The Nexus Between Economic Freedom and Tourist Arrivals: Evidence from Mediterranean Countries.” *Istanbul Journal of Economics / İstanbul İktisat Dergisi* 70 (2): 287–301. https://doi.org/10.26650/istjecon2020-825490.

Balli, Faruk, Hatice O. Balli, and Rosmy Jean Louis. 2016. “The Impacts of Immigrants and Institutions on Bilateral Tourism Flows.” *Tourism Management* 52: 221–29. https://doi.org/10.1016/j.tourman.2015.06.021.

Baltagi, B. 2005. “Econometric Analysis of Panel Data.” *Vasa*, 302.

Barro, R, and Xavier Sala-i-Martin. 2004. “Economic Growth, Chapter 2.” MIT Press, Cambridge.

Bulut, Umit, Emrah Kocak, and Courtney Suess. 2020. “The Effect of Freedom on International Tourism Demand: Empirical Evidence from the Top Eight Most Visited Countries.” *Tourism Economics* 26 (8): 1358–73. https://doi.org/10.1177/1354816619896957.

Chaisumpunsakul, Wipaporn, and Piriya Pholphirul. 2018. “Does International Trade Promote International Tourism Demand? Evidence from Thailand’s Trading Partners.” *Kasetsart Journal of Social Sciences* 39 (3): 393–400. https://doi.org/10.1016/j.kjss.2017.06.007.

Cheung, Yun Hsing, and Shrabani Saha. 2015. “Exploring the Nexus between Tourism Demand and Cultural Similarity.” *Tourism Analysis* 20 (2): 229–41. https://doi.org/10.3727/108354215X14265319207551.

Crawford, Duane W., and Geoffrey Godbey. 1987. “Reconceptualizing Barriers to Family Leisure.” *Leisure Sciences* 9 (2): 119–27. https://doi.org/10.1080/01490408709512151.

Das, Jayoti, and Cassandra E. DiRienzo. 2009. “Global Tourism Competitiveness and Freedom of the Press: A Nonlinear Relationship.” *Journal of Travel Research* 47 (4): 470–79. https://doi.org/10.1177/0047287508326535.

Davies, Brian, and Jean Mangan. 1992. “Family Expenditure on Hotels and Holidays.” *Annals of Tourism Research* 19 (4): 691–99. https://doi.org/10.1016/0160-7383(92)90061-S.

Easton, Stephen T, and Michael A Walker. 1997. “Income, Growth, and Economic Freedom.” *The American Economic Review* 87 (2): 328–32.

Fingleton, Bernard, and Enrique López-Bazo. 2006. “Empirical Growth Models with Spatial Effects.” *Papers in Regional Science* 85 (2): 177–98. https://doi.org/10.1111/j.1435-5957.2006.00074.x.

Forsyth, Peter, and Larry Dwyer. 2009. “Tourism Price Competitiveness.” In *The Travel & Tourism Competitiveness Report 2009*, 77–90.

Ghalia, Thaana, Jan Fidrmuc, Nahla Samargandi, and Kazi Sohag. 2019. “Institutional Quality, Political Risk and Tourism.” *Tourism Management Perspectives* 32: 100576. https://doi.org/10.1016/j.tmp.2019.100576.

Gholipour, Hassan Fereidouni, Reza Tajaddini, and Usama Al-mulali. 2014. “Does Personal Freedom Influence Outbound Tourism?” *Tourism Management* 41: 19–25. https://doi.org/10.1016/j.tourman.2013.08.010.

Gozgor, Giray, Chi Keung Marco Lau, Yan Zeng, and Zhibin Lin. 2019. “The Effectiveness of the Legal System and Inbound Tourism.” *Annals of Tourism Research* 76: 24–35. https://doi.org/10.1016/j.annals.2019.03.003.

Habibi, Fateh, and Khaled Ahmadzadeh. 2015. “Tourism Development , Trade Openness and Economic Growth : The Case of Malaysia.” *European Journal of Economica, Finance and Administrative Sciences* 78 (June): 130–37.

Hay, M. J., and K. E. McConnell. 1979. “An Analysis of Participation in Nonconsumptive Wildlife Recreation.” *Land Economics* 55 (4): 460–71. https://doi.org/10.2307/3145768.

Jiao, Shitai, Weijin Gong, Yanqiao Zheng, Xiaoqi Zhang, and Senlin Hu. 2019. “Spatial Spillover Effects and Tourism-Led Growth: An Analysis of Prefecture-Level Cities in China.” *Asia Pacific Journal of Tourism Research* 24 (7): 725–34. https://doi.org/10.1080/10941665.2019.1630454.

Kang, Sanghoon, Gyehee Lee, Jinwon Kim, and Deukhee Park. 2018. “Identifying the Spatial Structure of the Tourist Attraction System in South Korea Using GIS and Network Analysis: An Application of Anchor-Point Theory.” *Journal of Destination Marketing and Management* 9: 358–70. https://doi.org/10.1016/j.jdmm.2018.04.001.

Karimi, Mohammad Sharif, Mohsen Khezri, and Somayeh Razzaghi. 2021. “Impacts of Regional Conflicts on Tourism in Africa and the Middle East: A Spatial Panel Data Approach.” *Current Issues in Tourism,* 1–17. https://doi.org/10.1080/13683500.2021.1931054.

Kırant Yozcu, Özen. 2017. “Competitiveness of Istanbul as A Tourism Destination for Luxury Market.” *Journal of Tourismology* 3 (2): 2–13. https://doi.org/10.26650/jot.2017.3.2.0001.

Knack, Stephen, and Philip Keefer. 1995. “Institutions and Economic Performance: Cross-Country Tests Using Alternative Institutional Measures.” *Economics & Politics* 7 (3): 207–27. https://doi.org/10.1111/j.1468-0343.1995.tb00111.x.

Kubickova, Marketa. 2016. “The Role of Government in Tourism: Linking Competitiveness, Freedom, and Developing Economies.” *Czech Journal of Tourism* 5 (2): 73–92. https://doi.org/10.1515/cjot-2016-0005.

———. 2019. “The Impact of Government Policies on Destination Competitiveness in Developing Economies.” *Current Issues in Tourism* 22 (6): 619–42. https://doi.org/10.1080/13683500.2017.1296416.

Kulendran, N., and Kenneth Wilson. 2000. “Is There a Relationship between International Trade and International Travel?” *Applied Economics* 32 (8): 1001–9. https://doi.org/10.1080/000368400322057.

Lanouar, Charfeddine, and Mohamed Goaied. 2019. “Tourism, Terrorism and Political Violence in Tunisia: Evidence from Markov-Switching Models.” *Tourism Management* 70: 404–18. https://doi.org/10.1016/j.tourman.2018.09.002.

Larsen, Svein, Wibecke Brun, and Torvald Øgaard. 2009. “What Tourists Worry about - Construction of a Scale Measuring Tourist Worries.” *Tourism Management* 30 (2): 260–65. https://doi.org/10.1016/j.tourman.2008.06.004.

Lee, Choong Ki, Turgut Var, and Thomas W. Blaine. 1996. "Determinants of Inbound Tourist Expenditures." *Annals of Tourism Research* 23 (3): 527–42. https://doi.org/10.1016/0160-7383(95)00073-9.

LeSage, James, and R. Kelley Pace. 2009. *Introduction to Spatial Econometrics*. *Introduction to Spatial Econometrics*. Chapman and Hall/CRC. https://doi.org/10.1111/j.1467-985x.2010.00681_13.x.

Lim, Christine. 1997. "Review of International Tourism Demand Models." *Annals of Tourism Research* 24 (4): 835–49. https://doi.org/10.1016/s0160-7383(97)00049-2.

———. 2006. "A Survey of Tourism Demand Modelling Practice: Issues and Implications." *International Handbook on the Economics of Tourism*, 45–72. https://doi.org/10.4337/9781847201638.00008.

López, Fernando Sánchez. 2019. "Unemployment and Growth in the Tourism Sector in Mexico: Revisiting the Growth-Rate Version of Okun's Law." *Economies* 7 (3): 83. https://doi.org/10.3390/economies7030083.

Mamipour, Slab, and Fahimeh Abdi. 2017. "Spatial Spillover Effects of Tourism on Economic Growth in Iranian Provinces : Spatial β Convergence." *Journal of Tourism Planning and Development* 6 (22): 10–12.

Manzoor, Faiza, Longbao Wei, Muhammad Asif, Muhammad Zia Ul Haq, and Hafiz Ur Rehman. 2019. "The Contribution of Sustainable Tourism to Economic Growth and Employment in Pakistan." *International Journal of Environmental Research and Public Health* 16 (19): 3785. https://doi.org/10.3390/ijerph16193785.

Masiero, Lorenzo, and Juan L. Nicolau. 2012. "Tourism Market Segmentation Based on Price Sensitivity: Finding Similar Price Preferences on Tourism Activities." *Journal of Travel Research* 51 (4): 426–35. https://doi.org/10.1177/0047287511426339.

Meng, Weiqing, Lingying Xu, Beibei Hu, Jun Zhou, and Zhongliang Wang. 2017. "Reprint of: Quantifying Direct and Indirect Carbon Dioxide Emissions of the Chinese Tourism Industry." *Journal of Cleaner Production* 163: S401–9. https://doi.org/10.1016/j.jclepro.2016.03.177.

Mergoupis, Thanos, and Max Steuer. 2003. "Holiday Taking and Income." *Applied Economics* 35 (3): 269–84. https://doi.org/10.1080/00036840210143071.

Middleton, V. 1995. "Managing the Marketing Mix." *En Witt, SF y Moutinho, L., Eds., Tourism Marketing and Management Handbook, Prentice Hall, Hertfordshire*.

Miller, Terry, and Anthony B. Kim. 2017. "Index of Economic Freedom." *Washington: The Heritage Foundation*.

Morley, Clive L. 1992. "A Microeconomic Theory of International Tourism Demand." *Annals of Tourism Research* 19 (2): 250–67. https://doi.org/10.1016/0160-7383(92)90080-9.

Morrison, Alastair M. 1996. *Hospitality and Travel Marketing.* Delmar Publishers.

Öcal, Nadir, and Jülide Yildirim. 2010. "Regional Effects of Terrorism on Economic Growth in Turkey: A Geographically Weighted Regression Approach." *Journal of Peace Research* 47 (4): 477–89. https://doi.org/10.1177/0022343310364576.

Ozcan, Ceyhun Can, Murat Aslan, and Saban Nazlioglu. 2017. "Economic Freedom, Economic Growth and International Tourism for Post-Communist (Transition) Countries: A Panel Causality Analysis." *Theoretical and Applied Economics* XXIV (2): 75–98.

P. Pablo-Romero, María Del, and José A. Molina. 2013. "Tourism and Economic Growth: A Review of Empirical Literature." *Tourism Management Perspectives* 8: 28–41. https://doi.org/10.1016/j.tmp.2013.05.006.

Ponce, Pablo, Nathalie Aguirre-Padilla, Cristiana Oliveira, José Álvarez-García, and María de la Cruz del Río-Rama. 2020. “The Spatial Externalities of Tourism Activities in Poverty Reduction.” *Sustainability (Switzerland)* 12 (15): 6138. https://doi.org/10.3390/su12156138.

Rasool, Haroon, Shafat Maqbool, and Md. Tarique. 2021. “The Relationship between Tourism and Economic Growth among BRICS Countries: A Panel Cointegration Analysis.” *Future Business Journal* 7 (1): 1–11. https://doi.org/10.1186/s43093-020-00048-3.

Ritchie, Brent W. 2004. “Chaos, Crises and Disasters: A Strategic Approach to Crisis Management in the Tourism Industry.” *Tourism Management* 25 (6): 669–83. https://doi.org/10.1016/j.tourman.2003.09.004.

Romão, João, and Hisamitsu Saito. 2017. “A Spatial Analysis on the Determinants of Tourism Performance in Japanese Prefectures.” *Asia-Pacific Journal of Regional Science* 1 (1): 243–64. https://doi.org/10.1007/s41685-017-0038-0.

Rosselló, Jaume, Susanne Becken, and Maria Santana-Gallego. 2020. “The Effects of Natural Disasters on International Tourism: A Global Analysis.” *Tourism Management* 79: 104080. https://doi.org/10.1016/j.tourman.2020.104080.

Rugg, Donald. 1973. “The Choice of Journey Destination: A Theoretical and Empirical Analysis.” *The Review of Economics and Statistics* 55 (1): 64. https://doi.org/10.2307/1927995.

Saha, Shrabani, Jen Je Su, and Neil Campbell. 2017. “Does Political and Economic Freedom Matter for Inbound Tourism? A Cross-National Panel Data Estimation.” *Journal of Travel Research* 56 (2): 221–34. https://doi.org/10.1177/0047287515627028.

Saha, Shrabani, and Ghialy Yap. 2014. “The Moderation Effects of Political Instability and Terrorism on Tourism Development: A Cross-Country Panel Analysis.” *Journal of Travel Research* 53 (4): 509–21. https://doi.org/10.1177/0047287513496472.

Samitas, Aristeidis, Dimitrios Asteriou, Stathis Polyzos, and Dimitris Kenourgios. 2018. “Terrorist Incidents and Tourism Demand: Evidence from Greece.” *Tourism Management Perspectives* 25: 23–28. https://doi.org/10.1016/j.tmp.2017.10.005.

Seetanah, Boopen, Raja Sannassee, and Sawkut Rojid. 2015. “The Impact of Relative Prices on Tourism Demand for Mauritius: An Empirical Analysis.” *Development Southern Africa* 32 (3): 363–76. https://doi.org/10.1080/0376835X.2015.1010717.

Selimi, Nasir, Sc. Luljeta Sadiku, and Murat Sadiku. 2017. “The Impact of Tourism on Economic Growth in the Western Balkan Countries: An Empirical Analysis.” *International Journal of Business and Economic Sciences Applied Research* 10 (2): 19–25. https://doi.org/10.25103/ijbesar.102.02.

Shan, Jordan, and Ken Wilson. 2001. “Causality between Trade and Tourism: Empirical Evidence from China.” *Applied Economics Letters* 8 (4): 279–83.

Su, Yu Wen, and Hui Lin Lin. 2014. “Analysis of International Tourist Arrivals Worldwide: The Role of World Heritage Sites.” *Tourism Management* 40: 46–58. https://doi.org/10.1016/j.tourman.2013.04.005.

Suresh, K. G., Aviral Kumar Tiwari, Gazi Salah Uddin, and Ali Ahmed. 2018. “Tourism, Trade, and Economic Growth in India: A Frequency-Domain Analysis of Causality.” *Anatolia* 29 (3): 319–25. https://doi.org/10.1080/13032917.2017.1408025.

Tang, Chor Foon. 2018. “The Impacts of Governance and Institutions on Inbound Tourism Demand: Evidence from a Dynamic Panel Data Study.” *Asia Pacific Journal of Tourism Research* 23 (10): 1000–1007. https://doi.org/10.1080/10941665.2018.1513052.

Tian, Man-Wen, Shu-Rong Yan, Mohsen Khezri, Muhaamad Sharif Karimi, Mahnaz Mamghaderi, and Yousaf Ali Khan. 2021. "Regional Effects of the Renewable Energy Components on CO2 Emissions of Asia-Pacific Countries." *PloS One* 16 (10): e0256542. https://doi.org/10.1371/journal.pone.0256542.

Walsh, Richard G., Kun H. John, John R. McKean, and John G. Hof. 1992. "Effect of Price on Forecasts of Participation in Fish and Wildlife Recreation: An Aggregate Demand Model." *Journal of Leisure Research* 24 (2): 140–56. https://doi.org/10.1080/00222216.1992.11969882.

Yang, Yang, and Timothy Fik. 2014. "Spatial Effects in Regional Tourism Growth." *Annals of Tourism Research* 46: 144–62. https://doi.org/10.1016/j.annals.2014.03.007.

Yap, Ghialy, and Shrabani Saha. 2013. "Do Political Instability, Terrorism, and Corruption Have Deterring Effects on Tourism Development Even in the Presence of Unesco Heritage? A Cross-Country Panel Estimate." *Tourism Analysis* 18 (5): 587–99. https://doi.org/10.3727/108354213X13782245307911.

You, Wanhai, and Zhike Lv. 2018. "Spillover Effects of Economic Globalization on CO2 Emissions: A Spatial Panel Approach." *Energy Economics* 73: 248–57. https://doi.org/10.1016/j.eneco.2018.05.016.
