# Supplementary material for: Economic freedom index effects on inbound tourism in European countries: A spatial analysis
Source: PLoS One. 2025 Apr 1;20(4):e0316133. doi: 10.1371/journal.pone.0316133 (PMC11960978; doi:10.1371/journal.pone.0316133)
Supplement: Attachment — Submitted filename: Response to Reviewers.docx [file pone.0316133.s004.docx]

Manuscript PONE-D-23-19081

Title: Economic Freedom Index Effects on Tourist’s Arrivals in European Countries: A Spatial Panel Analysis

Journal: PLOS ONE

Dear Editor and Reviewers,

Thank you very much for the opportunity to revise and resubmit our manuscript. We appreciate the constructive and careful review of our manuscript by the reviewer and editor. We have attempted to address all issues raised in the review, either in the text itself and/or in our comments. We truly believe that the manuscript is in much better shape as a result of the changes we made in response to your critical comments. Of course, we are happy to revisit any of the issues raised by the reviewers and make additional changes to improve the overall quality of the work.

Below, we provide a point-by-point response to each of the concerns raised by the reviewer.

Reviewer’s comments:

**Reviewer #1:**

Authors have given considerable attention to the analysis of the data. However, it needs a better a structure. Writing style and gramma must be revised. Please refer to the comments made on manuscript.

Author: Thank you very much for your comment. In the revised version of the manuscript, we have attempted to address all these issues.

……………………………………………

**Reviewer #2:**

1. It would be beneficial to place greater emphasis on the crucial role of tourism at the global level, while also highlighting the interconnection between tourism and economic freedom. By incorporating these aspects into your introduction, you can create a more comprehensive and professional piece of writing.

Author: Thank you for your comment. The introduction has been rewritten. The highlighted sections have been added to the introduction according to the comments. Other text has been rewritten for better clarity.

2. While you have provided a good starting point, I would suggest focusing on specific theories that are particularly relevant to the topic. The following theories could be incorporated into your analysis: Tourism-led Economic Growth Theory, Input-Output Analysis, Tourism Satellite Account (TSA), Destination Competitiveness Theory, Economic Multiplier Theory, and Social Exchange Theory. These theories offer robust frameworks for understanding the economic impacts and benefits associated with the tourism industry. Furthermore, it is important to note that while these theories provide valuable insights, they are not exhaustive, and the selection of theories should be tailored to the specific context or region being examined. It would be beneficial to support your arguments by referring to empirical research, statistical analysis, and relevant case studies. This approach will enhance the validity and credibility of your findings, allowing you to effectively demonstrate the critical role of the tourism industry in driving economic prosperity.

Author: By examining all the theories proposed, we found the Destination Competitiveness Theory suitable for the purposes of the article. This theory is used in the manuscript.

3. The literature review appears to lack a clear logical flow, making it difficult for readers to follow the progression of ideas. Consider organizing the review in a more structured manner, such as by thematic categories or chronological order, to enhance its coherence and readability. It would be valuable to synthesize the findings and conclusions from the reviewed literature. Highlight the key themes, trends, and conflicting perspectives that emerge from the existing studies. This synthesis will not only provide a comprehensive overview but also help establish the rationale for your research and its contribution to the field.

Author: According to the points raised, we have made significant changes in the literature review section. We tried to apply the comments, especially the last paragraph has been added to highlight the key themes, trends, and conflicting perspectives.

4. Citations are consistently and accurately formatted throughout the literature review. Properly attributing the sources will enhance the credibility and professionalism of your work.

Author: All citations were rechecked and converted to journal format with Mendeley software.

5. To improve clarity, consider breaking down complex equations into smaller components and clearly defining each parameter used. Additionally, provide explanations or contextual information to help readers understand the purpose and meaning of the equations in the context of your study. Ensure that all parameters used in your methodology are described in a logical and easily understandable manner. Avoid assuming prior knowledge on the part of the reader. Provide clear explanations for each parameter, including their definitions.

Author: Explaining the methodology and modeling of spatial panel models is complex and extensive, and breaking and explaining the equations will lead to the expression of newer equations that need to be interpreted again. We have referenced studies related to the article's methodology for a more complete explanation. More important than understanding the equations is the interpretation of the estimated parameters of the variables, which is no different from other conventional panel models. However, if our explanation is still not convincing, we will try to delineate this more should we get another round of revisions.

**Reviewer #3:**

1. Abstract & keywords:

The abstract is poorly structured. Highlights of problem identification need to be included in the abstract. Highlighting the problem identification and stating the objectives of the study clearly with aid to understand the originality of the work.

Moreover, certain claims on the economic freedom index seemed to be vague.

Examples:
1. More importantly, the economic freedom index has a significant effect on tourism arrivals in Europe
• Need to clarify the nature and direction of the impacts of the index
2. While some components of economic freedom have marginal effects,
• This is again a vague statement and the author needs to be more specific in reporting his results even in the abstract
3. Our findings have meaningful implications for policymakers interested in sustainable tourism in European countries
• Better to state what these implications are in brief

Better to rewrite the abstract considering the above points.

Author: Thank you very much for your detailed comment. The abstract has been rewritten according to the points raised.

2. Introduction:
The introduction sets out a good platform for the study.

However, the identification of the research problem is not well justified.
Certain terms need to be defined in order to facilitate the understanding (ex: four significant categories of economic freedom by Heritage Foundation need to be defined. the rule of law, market openness, regulatory efficiency, and government size)

Better to expand the references rather than listing them all together in one place, so that the reader can understand which author has found out which relationship. (ex: few studies have examined the effects of press and personal freedom, civil liberties, and economic and political freedom, corruption, and economic freedom (Su and Lin 2014; Kubickova 2016; Saha, Su, and Campbell 2017; Tang 2018; Kubickova 2019; Bulut, Kocak, and Suess 2020; Öcal and Yildirim 2010)).

Author: In line with the comments raised by Reviewer 2 and these comments, the introduction has been rewritten considering. The highlighted sections have been added to the introduction according to the comments. Other text has been rewritten for better clarity.

3. Literature review:

A few issues are being identified in relation to the formatting and presentation of the section.
Certain citation formats need to be revised (Ex: first reference in the literature review section, Saha and Yap ((2014) on page no 05).

Author: All citations were rechecked and converted to journal format with Mendeley software.

4. Certain references are too old to be used and highlight the need to use recent articles.

Author: According to the points raised, the entire literature review section of the research was rewritten and some new articles are added. We tried to apply the comments, especially the last paragraph has been added to highlight the key themes, trends, and conflicting perspectives.

5. Conclusions:
Better to include a separate section for conclusions, implications and future research.

Author: this section is divided into the conclusions and implications sections.

6. References:

Referencing is satisfactory. However, need to revisit to the section as certain references cited in the text are not included in the list of references (ex: Heritage Foundation 2020) on page number 2 second paragraph).

Author: All citations were rechecked and converted to journal format with Mendeley software.

7. Language and formatting

The quality of English and general presentation is satisfactory.

While the expression is generally clear, there are a few issues in general word formatting, punctuation and grammar.

Author: The introduction and the background of the research were rewritten to fix possible writing errors.
