# Supplementary material for: Economic freedom index effects on inbound tourism in European countries: A spatial analysis
Source: PLoS One. 2025 Apr 1;20(4):e0316133. doi: 10.1371/journal.pone.0316133 (PMC11960978; doi:10.1371/journal.pone.0316133)
Supplement: Attachment — Submitted filename: Review.docx [file pone.0316133.s005.docx]

**Manuscript Number: PONE-D-23-19081R1**

**Title: Economic Freedom Index Effects on Inbound Tourism in European Countries: A Spatial Analysis**

1. Abstract & keywords:

Even though the second verson has been improved, it is better for the authors to emphasize on the significance or the contribution of this paper. At the moment, authors seems to spend too much on writing the results.

2. Introduction:

The revised verson has been improved much. However, there are still some minor issues. First of all, the brief introduction of the key issues are still missing. Authors should include the defintion in the first or second paragraph. Secondly, the research gap has been poorly emphasized in the introduction. Finally, authors might consider including the research objectives at the end of the introduction.

3. Literature review

While authors have included several studies, a logical focus is still missing. Authors should try to emphasize on the key point of each section, instead of just listing study.

4.Methodology

The structure of this section is quite strange.

The introduction of Destination Competitiveness Theory should be placed in the introduction or Literature review, not in the methodology. Second, objectives should be in the Introduction

Methodology should focus on how to collect and analyze the data.

5. Results:

6. Discussion

In the conclusion section, authors should not dicuss the data results. It should be in the Results section. Dicussion should focus on the findings of the study and its contribution.

There shoul be one separate section about limitation and future research (extend the last 3 sentences)

7. Language and formatting

While the language quality is acceptable, there are still minors grammar mistake. Please doublcheck.
