# Supplementary material for: Economic freedom index effects on inbound tourism in European countries: A spatial analysis
Source: PLoS One. 2025 Apr 1;20(4):e0316133. doi: 10.1371/journal.pone.0316133 (PMC11960978; doi:10.1371/journal.pone.0316133)
Supplement: Attachment — Submitted filename: comments2.docx [file pone.0316133.s006.docx]

Authors' responses to comments from the Editor and the Reviewer

Manuscript PONE-D-23-19081

Title: Economic Freedom Index Effects on Tourist's Arrivals in European Countries: A Spatial Panel Analysis

Journal: PLOS ONE

**Authors:**

We would like to thank the editor and the reviewer for taking time to carefully read the manuscript and providing very useful comments. We have accounted for all the changes suggested to the best of our abilities. Your insightful comments and suggestions have improved the quality of the manuscript significantly. We hope the revision is to your satisfaction.

**Reviewer #4:**

1- Abstract

The abstract of an empirical study should be more specific by reporting results in numbers rather than merely qualitative statements, as in the current revision; brief policy implications need to be included in this applied research and in specific contexts.

Authors: According to the reviewer's opinion, the Abstract was rewritten.

2- Methodology

- The choice of Destination Completeness Theory (DCT) should be further justified to replace others, such as the Tourism-led Growth Theory which seems to be more relevant to the topic and has already been mentioned in the literature review section. The Introduction of DCT should be put in the review section to make a more logical flow to build a framework.
- Consider organizing the methodology section to delve mainly into a framework or theoretical hypothesis (if any) that emerged from previous sections and provide explanations or justifications for the choice of the empirical strategy or specification of regression equations. This elaboration not only establishes the rationale for the research idea but also enhances the robustness of the estimated results.

Authors: According to the reviewer's recommendation, we investigated the Tourism-led Growth Theory. This theory talks about the effects of tourism on economic growth (while our study examines the effect of economic growth on tourism). Therefore, it is not compatible with the theoretical principles of our study. Due to the objections of other reviewers, we had to summarize this section to preserve the methodology section's comprehensiveness. Therefore, we transferred the initial materials to the literature review section.

3- Results/Data

- The database does not fully describe whether tourists come from only the EU or other countries. It should be from only the EU. A description of the countries list should also be added (maybe in the appendix) as the present data only code numbers instead of names of countries.

Authors: As highlighted in Table 1, total tourists arrival have been examined in this study. We put the list of countries on page 9 as a footnote according to the opinion of the honorable reviewer.

- The concepts and terms of economic freedom and its components need to be clearly defined by the authors rather than letting the reader guess, such as the Trade Openness. This can impact the study's outcomes since there are several different measures of trade openness (e.g., export, import, or the total of both to GDP).

Authors: Necessary corrections were made in Table 1. It should be noted that the indicators of economic freedom are explained after Table 2.

- The constructed variables and their descriptive statistics should be put in the methodology section together with the data section or in a separate section for better paper structure and to avoid disfunctioning the results section, which focuses on only the estimated results or main outcomes of the study.

Authors: We have modified the structure of the article to apply the reviewer's opinion

4- Estimation of the results

- This section causes much confusion and lacks consistency between paragraphs second and third, and within each one there is also inconsistency among sentences, mainly because of not indicating the terms home country and neighboring countries in many contexts. E.g., some of them are: "the increase in destination country prices positively affects tourist arrivals"; "As per theoretical predictions, an increase in the price level should increase the prices of goods and services in the destination countries, thereby enhancing the destination country's competitiveness"; "A review of Figure 2 reveals that for 170 countries in 2017, the relationship between price levels and tourist arrivals is a positive communication pattern." "Even an increase in neighboring countries' prices results in a flow of tourists to neighboring countries and a decline in entries."
- Since the data is not described clearly, as mentioned above, this statement needs to be fully justified and more specific: "The increase could be attributed to the arrival of tourists from neighboring countries, regional tourism industry development, or the overall increase in tourists in the developed world."

Authors: We read all the mentioned sections and other sections. The text has been completely rewritten and revised to remove any ambiguities. Please refer to pages 12, 15 and 16.

- The authors should use the usual way of presenting p-values for easier reading, such as *, **, and *** for different levels of significant statistics; several tables should be put in the appendix to spare space for other main results with further discussion.

Authors: A number of tables were moved to the appendix and asterisks were placed.

- Because the specification of regression models and their choices of variables are often subjective and there is a complicated nexus between each country's price level and tourist arrivals, the authors should provide a separate section or some validity tests and analyses on the robustness of the estimated results. Several sensitive tests may include using different subsets of the data (e.g., the European countries with and without the European Union); different development levels (e.g., GDP per capita); etc.

Author: A primary concern highlighted by fellow reviewers pertains to the extensive inclusion of computational tables within the article. Specifically, Tables 3 through 6 meticulously assess the array of diagnostic tests for optimal model selection prevalent in empirical studies. Furthermore, given the spatial nature of our panel data model, it entails a considerable quantity of estimated model coefficients. This complexity is compounded as the incorporation of additional variables inversely affects the model's degrees of freedom, introducing limitations to our analysis. Despite these methodological challenges, we have duly acknowledged the esteemed referee's insights in the concluding section, proposing them as avenues for future research endeavors.

5- Others

Some grammatical and typing/formating errors remain, such as: "under researched," "comprehensive comprehension," "Dependency Critique Theory (DCT)," "data," "Estimation of result." The whole paper should be rechecked for consistency in grammar, formatting, and structure.

Authors: We fixed these problems. Then, the whole text is checked by an editor. Modifications can be seen in the form of change.

**Reviewer #5:**

1. Abstract & keywords:

Even though the second verson has been improved, it is better for the authors to emphasize on the significance or the contribution of this paper. At the moment, authors seems to spend too much on writing the results.

Authors: According to the reviewers' opinions, the Abstract was rewritten.

2. Introduction:

The revised verson has been improved much. However, there are still some minor issues. First of all, the brief Introduction of the key issues are still missing. Authors should include the defintion in the first or second paragraph. Secondly, the research gap has been poorly emphasized in the Introduction. Finally, authors might consider including the research objectives at the end of the Introduction.

Authors: According to the reviewer's opinion, the Introduction was restructured entirely, and requested sections were added.

3. Literature review

While authors have included several studies, a logical focus is still missing. Authors should try to emphasize on the key point of each section, instead of just listing study.
Authors: We have summarized the key points at the end of this section.

4.Methodology
The structure of this section is quite strange.

The Introduction of Destination Competitiveness Theory should be placed in the Introduction or Literature review, not in the methodology. Second, objectives should be in the Introduction
Methodology should focus on how to collect and analyze the data.

Authors: we had to summarize this section to preserve the methodology section's comprehensiveness. Therefore, we transferred the initial materials to the literature review section.

6. Discussion

In the conclusion section, authors should not dicuss the data results. It should be in the Results section. Dicussion should focus on the findings of the study and its contribution.
There shoul be one separate section about limitation and future research (extend the last 3 sentences)
Authors: This problem has been fixed in the modified version, and future research is extended in the last paragraph.

7. Language and formatting

While the language quality is acceptable, there are still minors grammar mistake. Please doublcheck.

Authors: We fixed these problems. Then, the whole text is checked by an editor. Modifications can be seen in the form of change.

**Reviewer #6:**

I have read your previous notes and corrections. The changes you made are quite good and make your paper more interesting. However, I feel there is still something you need to complete, namely there needs to be a discussion section. This is considered very necessary because in the discussion section you will discuss reflections on all your findings, apart from that you can also argue the value of objectivity between theory and findings. Thank you.

Authors: Thanks for the reviewer's opinion. We have made some corrections in the conclusion section. We have considered this structure due to the compulsion to provide the opinion of all the honorable reviewers.
