# Supplementary material for: Economic freedom index effects on inbound tourism in European countries: A spatial analysis
Source: PLoS One. 2025 Apr 1;20(4):e0316133. doi: 10.1371/journal.pone.0316133 (PMC11960978; doi:10.1371/journal.pone.0316133)
Supplement: Attachment — Submitted filename: Review_auresp_3.docx [file pone.0316133.s007.docx]

**Author: Manuscript Number: PONE-D-23-19081R1**

**Title: Economic Freedom Index Effects on Inbound Tourism in European Countries: A Spatial Analysis**

1. Abstract & keywords:

Even though the second verson has been improved, it is better for the authors to emphasize on the significance or the contribution of this paper. At the moment, authors seems to spend too much on writing the results.

- Author: The abstract has been rewritten with more emphasis on the contribution of the article.

2. Introduction:

The revised verson has been improved much. However, there are still some minor issues. First of all, the brief introduction of the key issues are still missing:

- Author: The defects are completely fixed. Please see below for the exact address of each fixed problem.

Authors should include the defintion in the first or second paragraph.

- Author: The problem has been fixed. Page 1, first paragraph.

Secondly, the research gap has been poorly emphasized in the introduction.

- Author: The problem has been fixed. Page 3, last paragraph.

Finally, authors might consider including the research objectives at the end of the introduction.

- Author: The problem has been fixed. Page 4, first paragraph.

3. Literature review

While authors have included several studies, a logical focus is still missing. Authors should try to emphasize on the key point of each section, instead of just listing study.

- Author: The research literature was completely rewritten. We categorized the content, emphasizing on the key point of each section.

4.Methodology

The structure of this section is quite strange.

The introduction of Destination Competitiveness Theory should be placed in the introduction or Literature review, not in the methodology. Second, objectives should be in the Introduction

Methodology should focus on how to collect and analyze the data.

- Author: Irrelevant content was removed and defects were fixed. Please refer to the end of page 8.

5. Results:

6. Discussion

In the conclusion section, authors should not dicuss the data results. It should be in the Results section. Dicussion should focus on the findings of the study and its contribution.

There shoul be one separate section about limitation and future research (extend the last 3 sentences)

- Author: We have carefully reviewed this section and removed any material related to data analysis. Also, the research limitations section was added at the end along with the expanded recommendations. Please refer to page 18, last paragraph.

7. Language and formatting

While the language quality is acceptable, there are still minors grammar mistake. Please doublcheck.

- Author: We re-read the article and fixed every writing problem that we found.
