# Supplementary material for: Economic freedom index effects on inbound tourism in European countries: A spatial analysis
Source: PLoS One. 2025 Apr 1;20(4):e0316133. doi: 10.1371/journal.pone.0316133 (PMC11960978; doi:10.1371/journal.pone.0316133)
Supplement: Attachment — Submitted filename: PLOS ONE ECONOMIC FREEDOM AND TOURISM review report.docx [file pone.0316133.s008.docx]

1. I could not agree with your assertion that this is the first study that harnesses on a spatial analysis to unravel the relationship between economic freedom and tourism. For example, of existing studies in that direction see below:

*Ouchen, A., & Montargot, N. (2022). Non-spatial and spatial econometric analysis of tourism demand in a panel of countries around the world. Spatial Economic Analysis, 17(2), 262-283.*

*Xie, W., Li, H., & Yin, Y. (2021). Research on the spatial structure of the European union’s tourism economy and its effects. International Journal of Environmental Research and Public Health, 18(4), 1389.*

Based on this evidence, can you rephrase your statement and enhance your contribution.

2. The motivation of the study is not substantially justified given that your primary contribution is not grounded as there are numerous studies that have used the spatial approach, I am not convinced whether you have strongly justified your methodology and scope of study. Provide more information on the methods used by the existing studies in your literature review and tease out the gap you intend to fill. Since you failed to provide discernible evidence of the methodologies used in previous studies, it is difficult to accept that you are the first authors to use the methodology in your study.
3. Reconcile some statements in your conclusion and findings. For example;

“Furthermore, while the coefficients for six economic freedom indices are meaningless, the coefficients for the Trade Freedom Index, the Investment Freedom Index, the Property Rights Index, and the Government Integrity Index are significantly positive.” What does this statement mean? Insignificant or significant?

4. Can you justify why these findings are apparent? Provide some evidence-based rationale for these findings juxtaposing with previous studies.

“The neighboring countries' total economic freedom index demonstrates positive and significant effects. Furthermore, most economic freedom variables, such as the Business Freedom Index, the Investment Freedom Index, the Labor Freedom Index, the Trade Freedom Index, and the Government Integrity Index, exhibit a statistically significant and positive effect. However, neighboring countries' Monetary Freedom Index results in a decrease in the number of domestic arrivals. Among the various indicators, only improvements in the country's and neighboring countries' Investment Freedom Index and Government Integrity Index increase domestic arrivals. However, the Financial Freedom Index, the Tax Burden Index, and the Government Spending Index demonstrate no significant effect.”
