# Supplementary material for: Economic freedom index effects on inbound tourism in European countries: A spatial analysis
Source: PLoS One. 2025 Apr 1;20(4):e0316133. doi: 10.1371/journal.pone.0316133 (PMC11960978; doi:10.1371/journal.pone.0316133)
Supplement: Attachment — Submitted filename: Review_auresp_4.docx [file pone.0316133.s009.docx]

**Author: Manuscript Number: PONE-D-23-19081R1**

**Title: Economic Freedom Index Effects on Inbound Tourism in European Countries: A Spatial Analysis**

**Reviewer #7:**

- The written introduction needs to clearly show the practical context: Need more data..., lack of theoretical context

- Apply the theoretical base

- In order to solve the above problem, we added the theoretical base section titled Destination Competitiveness Theory (DCT) to the study. Please refer to page 7.
- Also, the contribution section of the article was modified to have a more practical and clear approach. Please refer to page 4.

- Old references need updating

- A number of new studies were also added to the article.

- The qualitative research part needs to be described in more detail such as: discussing the scale...

- Tables need deeper analysis.

- The estimation results section was rewritten for better clarity. See pages 12 and 13 for details.

- How should we compare the differences with other studies?

- The results of the study were compared with previous articles. Please refer to page 17 of the last paragraph.

- The implication is that the writing needs to be more focused.

- The Policy Implications results section was rewritten. Please refer to page 18 of the last paragraph and page 19 of the first paragraph.

**Reviewer #8:**

1. I could not agree with your assertion that this is the first study that harnesses on a spatial analysis to unravel the relationship between economic freedom and tourism. For example, of existing studies in that direction see below:

Ouchen, A., & Montargot, N. (2022). Non-spatial and spatial econometric analysis of tourism demand in a panel of countries around the world. Spatial Economic Analysis, 17(2), 262-283.

Xie, W., Li, H., & Yin, Y. (2021). Research on the spatial structure of the European union’s tourism economy and its effects. International Journal of Environmental Research and Public Health, 18(4), 1389.

Based on this evidence, can you rephrase your statement and enhance your contribution.

2. The motivation of the study is not substantially justified given that your primary contribution is not grounded as there are numerous studies that have used the spatial approach, I am not convinced whether you have strongly justified your methodology and scope of study. Provide more information on the methods used by the existing studies in your literature review and tease out the gap you intend to fill. Since you failed to provide discernible evidence of the methodologies used in previous studies, it is difficult to accept that you are the first authors to use the methodology in your study.

- Thank you for your comments. Of the two articles presented, only Ouchen and Montargot (2022) employed a spatial econometric approach. However, their model is incomplete and lacks the crucial table of marginal effects, which we have included as Table 9 in our article. This table is not generated by software like Stata; we had to use MATLAB programming to produce it. As stated in the contribution section of our article: "In essence, the contribution of this paper manifests across two principal domains: firstly, it comprehensively delves into the influence of economic freedom on the tourism industry, encompassing a broad array of economic freedom index components. Secondly, this study pioneers the adoption of a spatial panel data approach for examining the repercussions of economic freedom on tourism”, we did not claim that the spatial econometric method was used solely in our study. This article's primary innovation lies in investigating the effects of economic freedom indicators on the tourism industry. Even if we had conducted this study using other conventional econometric methods, it would represent a novel study. Obviously, we have made mistakes in clearly expressing the concepts, so we have tried to express the innovation of the study more clearly by referring to the studies mentioned by the honorable referee and other available studies to show that this methodology is used before. Please refer to page 4, first paragraph.

3. Reconcile some statements in your conclusion and findings. For example;

“Furthermore, while the coefficients for six economic freedom indices are meaningless, the coefficients for the Trade Freedom Index, the Investment Freedom Index, the Property Rights Index, and the Government Integrity Index are significantly positive.” What does this statement mean? Insignificant or significant?

- The problem has been fixed. Please refer to page 18, paragraph 2.

4. Can you justify why these findings are apparent? Provide some evidence-based rationale for these findings juxtaposing with previous studies.

“The neighboring countries' total economic freedom index demonstrates positive and significant effects. Furthermore, most economic freedom variables, such as the Business Freedom Index, the Investment Freedom Index, the Labor Freedom Index, the Trade Freedom Index, and the Government Integrity Index, exhibit a statistically significant and positive effect. However, neighboring countries' Monetary Freedom Index results in a decrease in the number of domestic arrivals. Among the various indicators, only improvements in the country's and neighboring countries' Investment Freedom Index and Government Integrity Index increase domestic arrivals. However, the Financial Freedom Index, the Tax Burden Index, and the Government Spending Index demonstrate no significant effect.”

- Explanations were added according to the opinion of the esteemed referee. Please refer to page 16 of the last paragraph, and page 17 of the first paragraph.
